# Associations between Self-Reported Physical Activity, Heel Ultrasound Parameters and Bone Health Measures in Post-Menopausal Women

**DOI:** 10.3390/ijerph16173177

**Published:** 2019-08-31

**Authors:** Bolaji Lilian Ilesanmi-Oyelere, Nicole C. Roy, Jane Coad, Marlena Cathorina Kruger

**Affiliations:** 1 School of Health Sciences, College of Health, Massey University, Tennent Drive, Palmerston North 4442, New Zealand; 2 Riddet Institute, Palmerston North 4442, New Zealand; 3 Food Nutrition & Health Team, AgResearch Grasslands, Palmerston North 4442, New Zealand; 4 High-Value Nutrition, National Science Challenge, Auckland 1142, New Zealand; 5 Department of Nutritional Science, School of Food and Advanced Technology, College of Sciences, Massey University, Palmerston North 4442, New Zealand

**Keywords:** activity energy expenditure, metabolic equivalent task, body composition, bone mineral density, ageing

## Abstract

Physical activity plays an important role in the maintenance of bone health from childhood through adulthood. This study aimed to explore the associations between self-reported physical activity (PA), activity energy expenditure (AEE), heel ultrasound parameters and bone health measures among older adult women. The AEE was estimated from the responses of questionnaires for 125 older adult women aged 54–81 years. The bone parameters were measured by dual energy x-ray absorptiometry (DXA) and heel ultrasound parameters by the heel quantitative ultrasound (QUS). This study showed that AEE and the metabolic equivalent task (MET) were positively correlated with the bone and heel ultrasound parameters. However, fat mass (FM) and fat percentage were negatively correlated with AEE and MET. In addition, the regression analysis showed that higher AEE was a strong predictor of a higher spine T-score (β = 0.212, *p* = 0.015), QUS T-score (β = 0.239, *p* = 0.011) and stiffness index (β = 0.240, *p* = 0.010) after adjusting for age, fat mass, lean mass, height and calcium intake. These results contribute to our understanding of the importance of physical activity in postmenopausal women by reiterating the benefits of physical activity for older adult women. Physical activity is an important tool for the prevention and management of osteoporosis.

## 1. Introduction

Globally, 61% of osteoporotic fractures occur in women. In addition, one in three women over the age of 50 worldwide experiences osteoporotic fracture [1]. The global burden of osteoporosis is increasing and its impact on the health system, economies and society will worsen [1]. Physical activity is a factor that can be modulated to reduce the risk of developing osteoporosis.

Physical activity is defined by the World Health Organization (WHO) as any bodily movement of the skeletal muscle which requires energy expenditure [2]. The composition and function of the human skeletal system is enhanced by physical activity, however this also deteriorates with age [3]. In addition, a lack of exercise and physical activity has been linked to bone loss and ultimately osteoporosis [4], the presence of which may cause falls that result in osteoporotic fractures in the elderly women.

Bone loss in women is accelerated with the onset of menopause, making this group the focus of attention worldwide in regards to osteoporosis. Regardless of the underlying factors in the aetiology of osteoporosis and bone loss, an adequate and appropriate exercise regime may be beneficial for the reduction or alleviation of bone loss in osteoporosis, thereby reducing the prevalence and incidence of fractures [5].

Studies have compared the efficiency and effectiveness of the quantitative ultrasound sonometry (QUS) and dual energy X-ray absorptiometry (DXA) in the measurement and prediction of bone status [6,7]. However, DXA is still widely known to be the gold standard for bone mineral density (BMD) measurement and evaluation [7]. In addition, age, race, sex, hormones and heredity as well as environmental features, such as nutrition and physical activity, have been reported as important determining factors for bone health [4,8]. Of these, nutrition and physical activity are the factors that can be easily be improved for bone health in a lifetime.

Furthermore, previous studies, including Cochrane reviews, have presented the effects of physical activity on bone strength across the lifespan [9,10], in children and adolescents [11] as well as in women, especially for postmenopausal women [12,13,14]. A study from Italy on the effectiveness of physical activity types on BMD in osteoporotic patients reported progressive resistance strength training of the lower limbs to be most effective for the neck of the femur, while a multicomponent training programme was suggested for spine BMD intervention [15].

Neilson et al. defined activity energy expenditure (AEE) as a modifiable component of total energy expenditure (TEE) derived from both volitional and non-volitional activities [16]. Total energy expenditure comprises multiple components, including physical activity energy expenditure (PAEE), resting energy expenditure (REE), and the thermic effect of food (TEF) [17]. This study estimated AEE from the metabolic equivalent task (MET). The MET is used to express the intensity and energy expenditure of activities among people of varying weight. Furthermore, actual energy expenditure (measured in kilocalories or kilojoules) during a particular activity depends on the individual’s total body mass. The energy cost of the same type of activity is different for individuals of different weight [18].

The impact of physical activity has been used to alleviate several obesity-related diseases and the overall burden of diseases in men and women alike [19]. Although studies have investigated the effects of long-term physical activity, or a physically active lifestyle, on adult bone health parameters, to the authors’ knowledge, no study has reported on the relationship between recent short-term physical activity and measures of bone health. A specific tool to achieve this is the interviewer-administered New Zealand Physical Activity Questionnaire—short form (NZPAQ-SF). The specific aim of this research was to investigate the relationship between self-reported AEE, MET and QUS heel ultrasound and DXA outputs in a cohort of postmenopausal women. It was hypothesized that the participants with higher reported physical activity (PA) measured by AEE and MET have higher BMD, that is, a positive association.

## 2. Materials and Methods

### 2.1. Study Design

A total of 125 postmenopausal women aged between 54 and 81 years participated in the BugsnBones study that took place in the Human Nutrition Research Unit of Massey University, Palmerston North campus from June to December 2017. The majority (*n* = 124, 99.2%) of the participants were New Zealanders of European descent, who self-reported their ethnicity as New Zealand Europeans or Pakeha’ The sample size of the cross-sectional study was calculated using G*Power software version 3.0.10 (Heinrich-Heine-Universität Düsseldorf, Düsseldorf, Germany) and eighty-eight subjects were required for a 95% power and an alpha of 5% for the *t*-test. This study estimated a total of one hundred and fifty participants based on the osteoporosis incidence ratio of 3:1 in women. Two subjects were excluded from the study, one due to a ketogenic diet and the other due to health conditions. The subjects were recruited by advertisement on campus, the Whanganui Chronicle and by using a recruitment agency; Trial Facts (https://trialfacts.com/). The inclusion criteria were confirmed as menopause of at least 5 years based on no menstruation. The exclusion criteria were the presence of any systemic disease, food intolerances which affect the gastrointestinal tract, smoking and high intake of alcohol. The subjects with significant weight loss or weight gain within the past year were also excluded. All subjects were free living and apparently healthy. Written informed consent was obtained from the subjects before commencing data collection. This study was approved by the Massey University Human Ethics Committee: Southern A, Application 17/17. The study was registered with the Australian New Zealand Clinical Trials Registry (ANZCTR) with the number ACTRN12617000802303.

### 2.2. Anthropometric Measurements of the Subjects

The body weight of participants was measured using the Detecto 437 eye-level weigh beam physician scale to the nearest 0.2 kg and standing height was measured using a wall mounted rolled stadiometer to the nearest 0.1 cm wearing light clothes and no shoes on. Body mass index (BMI) was calculated as the weight divided by height squared (kg/m^2^). The waist to hip ratio was determined by measuring the waist and hip circumference to the nearest 0.1 cm using a non-stretchable tape. The waist to hip ratio was used as a marker of abdominal obesity.

### 2.3. Physical Activity Questionnaire

The significance of physical activity was investigated amongst postmenopausal women in the Manawatu-Wanganui region of New Zealand [20] using the NZPAQ-SF to support and substantiate our previous article’s findings of the same study. The previous study investigated lean body mass in relation to bone health [20]. The authors performed more in-depth analyses of the data collected from the larger BugsnBones study.

Physical activity was assessed using the NZPAQ-SF [21], which has previously been validated by Boon and colleagues [22]. Physical activities were quantified by MET-minutes/day and AEE-Cal/day which was calculated by using the scoring protocol of the international physical activity questionnaire (IPAQ) for continuous scores. The MET values and the formula for computation of MET-minutes were as follows [23].

- Walking MET-minutes/week at work = 3.3 * walking minutes * walking days at work.
- Moderate MET-minutes/week at work = 4.0 * moderate-intensity activity minutes * moderate intensity days at work.
- Vigorous MET-minutes/week at work = 8.0 * vigorous-intensity activity minutes * vigorous intensity days at work Total Work MET-minutes/week = sum of Walking + Moderate + Vigorous MET-minutes/week scores at work.
- Total PA MET-minutes/week = sum of walking + moderate + vigorous MET-minutes/week scores.

The (NZPAQ-MVPA) was derived from the total weekly minutes of vigorous + moderate activity in bouts of ≥10 min excluding walking.

### 2.4. DXA and QUS Measurements

All body composition measurements, fat mass (FM), lean mass (LM) and fat percentage were measured and analysed using the Hologic QDR series Discovery A, Bone densitometry DXA, USA. The participants’ BMD was measured at the femoral neck (FN), lumbar spine (LS) [L1–L4], trochanter, Ward’s triangle and total hip. The DXA machine was calibrated every morning and at the end of each day for all measurements. The in vivo reproducibility of the coefficient of variation ranged between 0.34–0.70% for all measured sites. The reported LS BMD values were calculated as the means of four measured values from L1–L4. The Apex System Software version 4.5.3 (Hologic Inc., Bedford, MA, USA) was used for analysing the DXA scans. Osteoporosis was defined as a T score ≤ −2.5 and osteopenia as a T score between −1.0 and −2.5, according to the WHO criteria [24].

The Quantitative ultrasound (QUS) of the non-dominant heel scan was measured using the GE Lunar Achilles II Portable Bone Densitometer (Hologic Inc., Northborough, MA, USA). The outputs included the stiffness index, the broadband ultrasound attenuation (BUA measured in dB/MHz), the speed of sound (SOS measured in m/s), the QUS T-score and QUS Z-score.

### 2.5. Calcium Intake Assessment

The participants’ calcium intake assessment was investigated with a food frequency questionnaire (FFQ). The 108-item FFQ was used to collect information on the frequency of the participants’ food and beverage intake utilizing household measures for servings. The Food works 9 version software was used to analyze the participants’ calcium intake data.

### 2.6. Statistical Analysis

IBM SPSS version 25 (IBM Company, Armonk, NY, USA) was used for all statistical analyses. The values of all variables’ regional sites were presented as the mean ± standard deviation. The analysis of covariance (ANCOVA) was used to evaluate the differences in physical activity measures according to the hip osteoporotic groups (osteopenic and osteoporotic women) versus healthy. The partial correlation analyses of the regional sites and QUS parameters with AEE and MET were performed and were adjusted for age, weight and height. The regression analysis of QUS parameters and skeletal site measurements by AEE was conducted and confounders such as age, fat mass, lean mass, height and dietary calcium intake were regressed into the model. The outcome variables used were the T-scores of skeletal sites as well as body composition measures. The regression analysis was used to obtain the determinants and predictors for the outcome variables, showing the relationship between these variables and bone health indicators. All *p*-values were reported significant at 0.05 or less.

## 3. Results

As shown in Table 1, the average weight of the subjects was 69.3 kg with a standard deviation of 11.2 kg. The average AEE calculated was 479 Cal/day from the physical activities recorded.

The classification into osteoporotic status based on the hip revealed that healthy individuals were significantly different than the osteoporotic groups (F_2119_ = 5.77, *p* = 0.004). The significant differences were also observed between the groups for age and weight according to the hip osteoporotic status (F_1119_ = 5.97, *p* = 0.016) and (F_1119_ = 33.01, *p* ≤ 0.001) respectively but not for height (Table 2).

Taking into account the effects of age, weight and height, the partial correlation coefficients are as shown below. The effects of AEE and MET were only significant for femoral neck BMD and the T-score. Similarly, the relationship between the stiffness index and measures of PA measures were significant. The positive correlations were observed between LM and AEE (r = 0.140, *p* = 0.130) and MET (r =0.177, *p* = 0.056) were not significant. However, there were negative correlations between AEE and fat percentage (r = −0.160, *p* = 0.083), likewise MET and fat percentage (r = −0.229, *p* = 0.013). The strongest correlations were found between the PA measures and the QUS parameters (Table 3).

To assess the extent of the impact of body composition, the calcium intake and physical activity on QUS and bone measures, the regression analysis was performed. Table 4 displays age, fat mass, lean mass, height, dietary calcium intake and AEE (Cal/day) as a predictor for bone health measures and QUS parameters. The dependent variables included were regional site T-scores, QUS outputs and body composition. The regression analysis showed that higher AEE was a strong predictor of higher spine T-score (β = 0.212, *p* = 0.015), QUS T-score (β = 0.239, *p* = 0.011) and the stiffness index (β = 0.240, *p* = 0.010) after adjusting for age, weight, height and calcium intake. However, AEE was not significantly associated with the hip or femoral neck T-scores. As shown in Table 4, the regression analysis shows the relationship between the dependent variables; spine, hip, femoral neck and QUS T-scores, lean mass and fat mass, and AEE.

## 4. Discussion

The study sought to determine the extent to which self-reported physical activity (calculated as AEE) predicts bone, QUS parameters and body composition for New Zealand European postmenopausal women. This work provided detailed anthropometry measurements, ANCOVA comparison of healthy and osteoporotic groups according to their self-reported physical activity as well as the partial correlation analyses for bone and physical activity parameters. Comparisons between the groups revealed higher AEE and MET for the healthy groups than the osteoporotic. Although not significant, the positive partial correlations were observed between the physical activity measures (calculated AEE and MET) and bone health parameters. The partial correlations between physical activity measures and the FM and fat percentage were negative.

Furthermore, the regression analysis showed the extent to which self-reported AEE may determine the bone health, QUS and body composition status in older women after adjusting for confounders. In keeping with other studies [4,25], AEE is significantly positively associated with the spine T-score, QUS T-score, and the stiffness index but no significant association was found with the femoral neck and hip T-scores in postmenopausal women. The results of this paper indicate that higher AEE is strongly associated with higher spine T-scores and QUS parameters among older adult women.

The relationship between self-reported physical activity and bone health was especially more pronounced for the QUS of the heel which may be because the heel bears a higher load in comparison to other parts of the body. The partial correlations also revealed significant positive associations between the femoral neck and PA measures. This is especially important since fragility fractures mainly severely occur at the femoral neck of the hip. The data from this study showed two women reported no physical activity in the week prior to the study date. One was osteopenic and the other healthy, based on the spine classification.

Similar studies have been conducted by other researchers looking at the relationship between self-reported and measured/monitored AEE and bone health [8]. A study by Milliken et al. [26] investigated the effects of exercise on BMD in postmenopausal women. The form of physical activity used was a one-year supervised exercise training program, measured as the total amount of weight lifted for the duration of the program. The results of this study indicated a statistically significant positive effect of exercise on trochanter and FN-BMD, but not that of LS. The authors found that the cumulative weight lifted statistically significantly predicted BMD of the trochanter [27]. Nevertheless, the present study differs from that of Milliken et al. due to the fact that there was not a relationship between FN-BMD and exercise. This may be as a result of the sample size. However, the lumbar spine BMD and the T-score was statistically significantly associated with AEE.

Body weight, age, sex and the duration of sleep influence AEE. Therefore, the individuals who perform the same amount of activity and weigh more, expend more energy than those who weigh less [28]. This study found negative correlations between FM, fat percentage and self-reported AEE. Furthermore, the result of this study is similar to previously reported papers which linked physical inactivity with body weight and obesity [19,25,29]. This study found physical inactivity was associated with a higher percentage of body fat and FM.

In summary, these results show that physical activity is associated with higher bone mass, the stiffness index, lean body mass and a lower percentage of fat and FM. The strengths of this research in terms of the self-reported NZPAQ include its low-cost methodology, the fact that recall does not alter behaviour and its suitability for the population being studied. The limitations of this research include its cross-sectional nature. Therefore, problems of causal associations exist, and the use of self-reported physical activity from the questionnaires for estimating AEE. However, the MET has been reported as an acceptable methodology for measuring physical activity [4]. This study needs to be replicated in other ethnicities and communities to be representative of the general population.

## 5. Conclusions

In conclusion, this study evaluated the self-reported AEE as a predictor of bone and QUS status, the stiffness index and body composition. AEE showed a strong positive association with bone health measures. This supports the aim of this research which shows that PA is positively related to bone health status in older adult women and negatively associated with fat percentage and FM. This study is the first to use the NZPAQ-SF to investigate the relationship between self-reported physical activity and bone health.

## Figures and Tables

**Table 1:** The subjects’ baseline characteristics and anthropometric variables.

Parameters	(*n* = 125)
	Mean ± SD
Age (years)	62.6 ± 4.5
Weight (kg)	69.3 ± 11.2
Height (cm)	162.3 ± 5.3
BMI (kg/m^2^)	26.3 ± 4.2
WC (cm)	80.8 ± 10.8
HC (cm)	99.3 ± 7.6
WH ratio	0.8 ± 0.1
Spine BMC (g)	54.2 ± 11.4
Spine BMD (g/cm^2^)	0.94 ± 0.15
Spine T-score	−0.9 ± 1.4
Femoral neck BMC (g)	3.6 ± 0.5
Femoral neck BMD (g/cm^2^)	0.7 ± 0.1
Femoral neck T-score	−1.2 ± 0.9
Hip BMC (g)	29.9 ± 5.1
Hip BMD (g/cm^2^)	0.9 ± 0.1
Hip T-score	−0.7 ± 1.0
Whole body total fat mass (kg)	29.4 ± 8.3
Whole body total lean mass (kg)	40.6 ± 4.5
Whole body total fat %	41.2 ± 6.5
Stiffness index	88.9 ± 13.6
BUA (dB/MHz)	110.7 ± 11.2
SOS (m/s)	1554.4 ± 31.0
QUS T-score	−0.7 ± 0.9
QUS Z-score	0.8 ± 0.8
AEE (Cal/day)	479.1 ± 772.7
MVPA MET-minutes/week	1644.0 ± 1970.4
Walk MET-minutes/week	624.8 ± 839.1
Moderate MET-minutes/week	918.2 ± 1028.3
Vigorous MET-minutes/week	725.8 ± 1414.4
Total MET-minutes/week	2268.8 ± 2374.5
Calcium intake (mg)	1263.5 ± 851.4

SD = standard deviation; BMI = body mass index; WC = waist circumference; HC = hip circumference; WH = waist to hip; BMC = bone mineral content; BMD = bone mineral density; BUA = broadband ultrasound attenuation; SOS = speed of sound; QUS = Quantitative Ultrasound; AEE = activity energy expenditure; MET = metabolic equivalent of task.

**Table 2:** Summary of ANCOVA according to hip osteoporotic status.

ANCOVA Based on the Hip Osteoporotic Status					
Parameters	Sum of Squares ^1^	Degrees of Freedom	Mean Square	F	*p*-Value
Tertiles of Total METs	2.033	2	1.017	5.769	0.004
Age (years)	1.052	1	1.052	5.967	0.016
Weight (kg)	5.818	1	5.818	33.013	<0.001
Height (cm)	0.058	1	0.058	0.327	0.568
Error	20.972	119	0.176		
Total	50.000	125			

^1^ R Squared = 0.301.

**Table 3:** The partial correlation coefficients of bone health and quantitative ultrasound (QUS) parameters with PA measures adjusted for age, weight and height.

Bone Health Equivalent Parameters	Activity Energy Expenditure (Cal/Day)	Metabolic of Task (Minutes/Week)
Spine BMD (g/cm^2^)	0.110 ^ns^	0.106 ^ns^
Spine T-score	0.104 ^ns^	0.100 ^ns^
Femoral neck BMD (g/cm^2^)	0.208 *	0.212 *
Femoral neck T-score	0.206 *	0.210 *
Hip BMD (g/cm^2^)	0.144 ^ns^	0.147 ^ns^
Hip T-score	0.121 ^ns^	0.123 ^ns^
Stiffness Index	0.312 ***	0.321 ***
BUA (dB/MHz)	0.162 ^ns^	0.160 ^ns^
SOS (m/s)	0.341 ***	0.356 ***
QUS T-score	0.313 ***	0.323 ***
QUS Z-score	0.315 ***	0.323 ***
Fat mass (kg)	−0.134 ^ns^	−0.170 ^ns^
Lean mass (kg)	0.140 ^ns^	0.177 ^ns^
Fat %	−0.160 ^ns^	−0.229 *

* *p* < 0.05; *** *p* < 0.001 (two tailed). BMD = bone mineral density; BUA = broadband ultrasound attenuation; SOS = speed of sound; QUS = Quantitative Ultrasound; ns = not significant.

**Table 4:** Regression analysis of bone health, QUS parameters and body composition.

Parameters	β	CI	R^2^	*p*
Spine T-score			0.341	
Age (years)	0.020	−0.043, 0.055		0.803
Fat mass (kg)	0.293	0.016, 0.079		0.003
Lean mass (kg)	0.292	0.019, 0.159		0.013
Height (cm)	−0.155	−0.092, 0.010		0.116
Calcium intake (mg)	0.223	0.0001, 0.001		0.006
AEE (Cal/day)	0.212	0.000,0.001		0.015
Femoral neck T-score			0.367	
Age (years)	−0.241	−0.079, −0.017		0.003
Fat mass (kg)	0.142	−0.006, 0.036		0.150
Lean mass (kg)	0.378	0.029, 0.118		0.001
Height (cm)	0.001	−0.033, 0.033		0.991
Calcium intake (mg)	0.068	0.000, 0.0002		0.397
AEE (Cal/day)	0.156	−0.000, 0.001		0.060
Hip T-score			0.338	
Age (years)	−0.226	−0.084, −0.015		0.006
Fat mass (kg)	0.254	0.007, 0.051		0.011
Lean mass (kg)	0.348	0.026, 0.124		0.003
Height (cm)	−0.037	−0.043, 0.029		0.710
Calcium intake (mg)	0.018	−0.0002, 0.0002		0.821
AEE (Cal/day)	0.015	−0.0002, 0.0002		0.863
Whole body BMD			0.198	
Age (years)	−0.133	−0.008, 0.001		0.136
Fat mass (kg)	0.034	−0.002, 0.003		0.754
Lean mass (kg)	0.295	0.001, 0.013		0.022
Height (cm)	0.025	−0.004, 0.005		0.815
Calcium intake (mg)	0.204	0.000, 0.000		0.022
AEE (Cal/day)	0.015	−0.000, 0.000		0.873
QUS T-score			0.245	
Age (years)	−0.197	−0.071, −0.005		0.024
Fat mass (kg)	0.026	−0.018, 0.024		0.805
Lean mass (kg)	0.312	0.013, 0.107		0.013
Height (cm)	−0.031	−0.040, 0.029		0.771
Calcium intake (mg)	−0.102	−0.000, 0.0001		0.234
AEE (Cal/day)	0.239	0.000, 0.001		0.011
Stiffness Index			0.246	
Age (years)	−0.196	−1.133, −0.083		0.024
Fat mass (kg)	0.022	−0.301, 0.373		0.832
Lean mass (kg)	0.314	0.214, 1.999		0.012
Height (cm)	−0.031	−0.633, 0.469		0.769
Calcium intake (mg)	−0.098	−0.004, 0.001		0.252
AEE (Cal/day)	0.240	0.001, 0.007		0.010

BMD = bone mineral density; QUS = Quantitative Ultrasound; AEE = activity energy expenditure; CI = 95% confidence interval. Model predictors are age, fat mass, lean mass, height, calcium intake and AEE.
